# Psychiatric Comorbidities and Survival After Lobectomy for Stage I Non-Small Cell Lung Cancer

**DOI:** 10.1016/j.atssr.2024.07.013

**Published:** 2024-07-29

**Authors:** Jesse York, James O’Toole, Andrea Makowski, Alyssa Woodward, Rebecca Suk, Roman Petrov, Charles Bakhos, Doraid Jarrar, Sai Yendamuri

**Affiliations:** 1 Texas Tech University Health Sciences Center School of Medicine, Lubbock, Texas; 2 St. George’s University School of Medicine, True Blue, Grenada; 3 Drexel University College of Medicine, Philadelphia, Pennsylvania; 4 University of Nevada, Reno School of Medicine, Reno, Nevada; 5 Alabama College of Osteopathic Medicine, Dothan, Alabama; 6 Department of Surgery, University of Texas Medical Branch John Sealy School of Medicine, Galveston, Texas; 7 Department of Surgery, Lewis Katz School of Medicine, Temple University, Philadelphia, Pennsylvania; 8 Department of Surgery, Perelman School of Medicine at the University of Pennsylvania, Philadelphia, Pennsylvania; 9 Department of Thoracic Surgery, Roswell Park Comprehensive Cancer Center, Buffalo, New York

## Abstract

**Background:**

The association between psychiatric comorbidities (PCs) and long-term survival after lobectomy for early-stage non-small cell lung cancer is unknown. We sought to investigate this relationship using the Surveillance, Epidemiology, & End-Results (SEER)-Medicare registry.

**Methods:**

Data for all patients in the SEER-Medicare registry who underwent lobectomy for stage I non-small cell lung cancer from 2007 to 2014 were included. Those older than 80 years at time of diagnosis, with multiple cancers, or histology other than adenocarcinoma or squamous cell carcinoma were excluded. Patients diagnosed with depression, anxiety, bipolar disorder, schizophrenia, other (non-schizophrenic) psychotic disorders, unspecified mood disorder, attention-deficit/hyperactivity disorder, alcohol use disorder, or substance use disorder prior to lung cancer diagnosis were considered to have a PC. Survival of patients with PC was compared to controls using univariable and multivariable analysis adjusting for age, sex, race, stage (IA/IB), histology, surgical approach, and Charlson Comorbidity Index.

**Results:**

Of 5516 patients, 1369 (24.8%) had PCs. Patients with PCs were more likely to be younger (*P* < .001), female (*P* < .001), white (*P* < .001), and have stage IA cancer (*P* < .001). PC was associated with shorter survival (median, 2478 vs 2820 days; *P* = .002). Multivariable analysis retained PC in the final model, with a hazard ratio of 1.10 (*P* < .001). Subgroup analysis revealed that this survival difference was driven by differences in survival of patients with schizophrenia (*P* < .001) and alcohol use disorder (*P* = .006).

**Conclusions:**

Select psychiatric comorbidities are associated with reduced survival after lobectomy for early-stage non-small cell lung cancer.

## Visual Abstract

**Figure:**
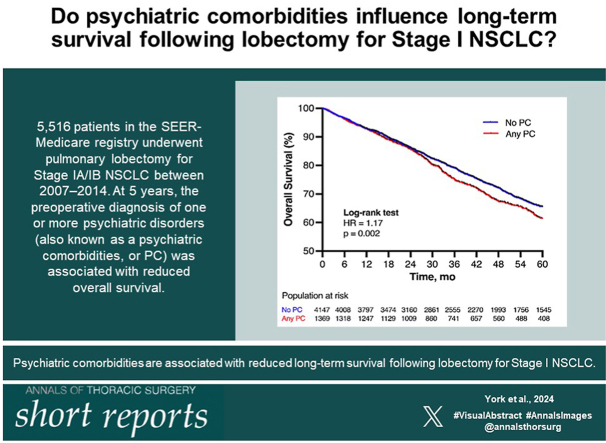

**In Short**

▪ Select psychiatric comorbidities are associated with reduced 5-year overall survival after lobectomy for stage I NSCLC.
▪ After adjusting for confounding variables, schizophrenia and alcohol use disorder were significantly and independently associated with mortality in the 5-year postoperative period.

Previous studies have investigated the relationship between preexisting psychiatric conditions (also referred to as psychiatric comorbidities, or PCs) and outcomes after surgery for non-small cell lung cancer (NSCLC).^1^, ^2^, ^3^ The principal aim of this study is to elucidate the association between psychiatric comorbidities and long-term overall survival in patients who undergo pulmonary lobectomy for early-stage NSCLC.

## Patients and Methods

### Patients

This study examined patients in the Surveillance, Epidemiology, and End Results (SEER)-Medicare database diagnosed with stage I (American Joint Committee on Cancer, *Cancer Staging Manual*, 6th edition) primary adenocarcinoma or squamous cell carcinoma of the lung between 2007 and 2014. Follow-up data from 2007 to 2019 were included, with survival outcomes assessed until death or up to 5 years postsurgery.

Stage I NSCLC was chosen to create a uniform cohort of patients whose survival is predominantly defined by surgical intervention rather than clinical stage or response to adjuvant therapies. Restriction to the adenocarcinoma and squamous cell carcinoma subtypes was performed to limit biological variability while retaining generalizability to a broad NSCLC population. Only patients who had NSCLC as the single primary malignancy in their lifetime were included to ensure that other cancers did not influence survival data. In addition, only patients aged 65-80 years at the time of NSCLC diagnosis were included. This age range was selected to ensure Medicare eligibility and to avoid confounding by noncancer mortality.

Patients who received radiation, surgery other than lobectomy as the primary NSCLC treatment, died within 3 months of diagnosis, lacked complete and sole Medicare coverage during the 12 months before and 1 month after diagnosis, or had unknown dates of lobectomy or diagnosis were excluded. Individuals were deemed to have PCs only if their Medicare part B, part D, or outpatient claims data noted such diagnoses within 1 year prior to NSCLC diagnosis. Diagnoses were identified using International Classification of Disease*,* 9th revision (ICD-9) codes in the 290-319 series (“Mental Disorders”) or ICD-10 codes in the F01-99 series (“Mental, Behavioral and Neurodevelopmental”) that the Mental Health Research Network uses for these 9 core diagnosis classes of psychiatric conditions: alcohol use disorder, substance use disorder, schizophrenia, nonschizophrenic psychotic disorders, bipolar I/II disorder, major depressive disorder, nonspecified affective and mood disorders, anxiety disorder, and attention-deficit/hyperactivity disorder.^4^

### Statistical Analysis

Two-group comparisons were performed with χ^2^ and Welch’s *t* tests for categorical and continuous variables, respectively. Overall survival in days after lobectomy was used for survival analyses with Kaplan-Meier method and Cox proportional hazards regression. The Cox proportional hazards models utilized backward stepwise consideration of age, sex, race, cancer stage (IA/IB), cancer histology, surgical approach, Charlson Comorbidity Index, and PC diagnosis in multivariable regression. Significance level was set at α = 0.05. Data lookup, filtering, and summarization were performed using R, Version 4.3 (The R Foundation for Statistical Computing). Statistical analyses were conducted using IBM SPSS Statistics, Version 29.0.

## Results

**Figure 1:**
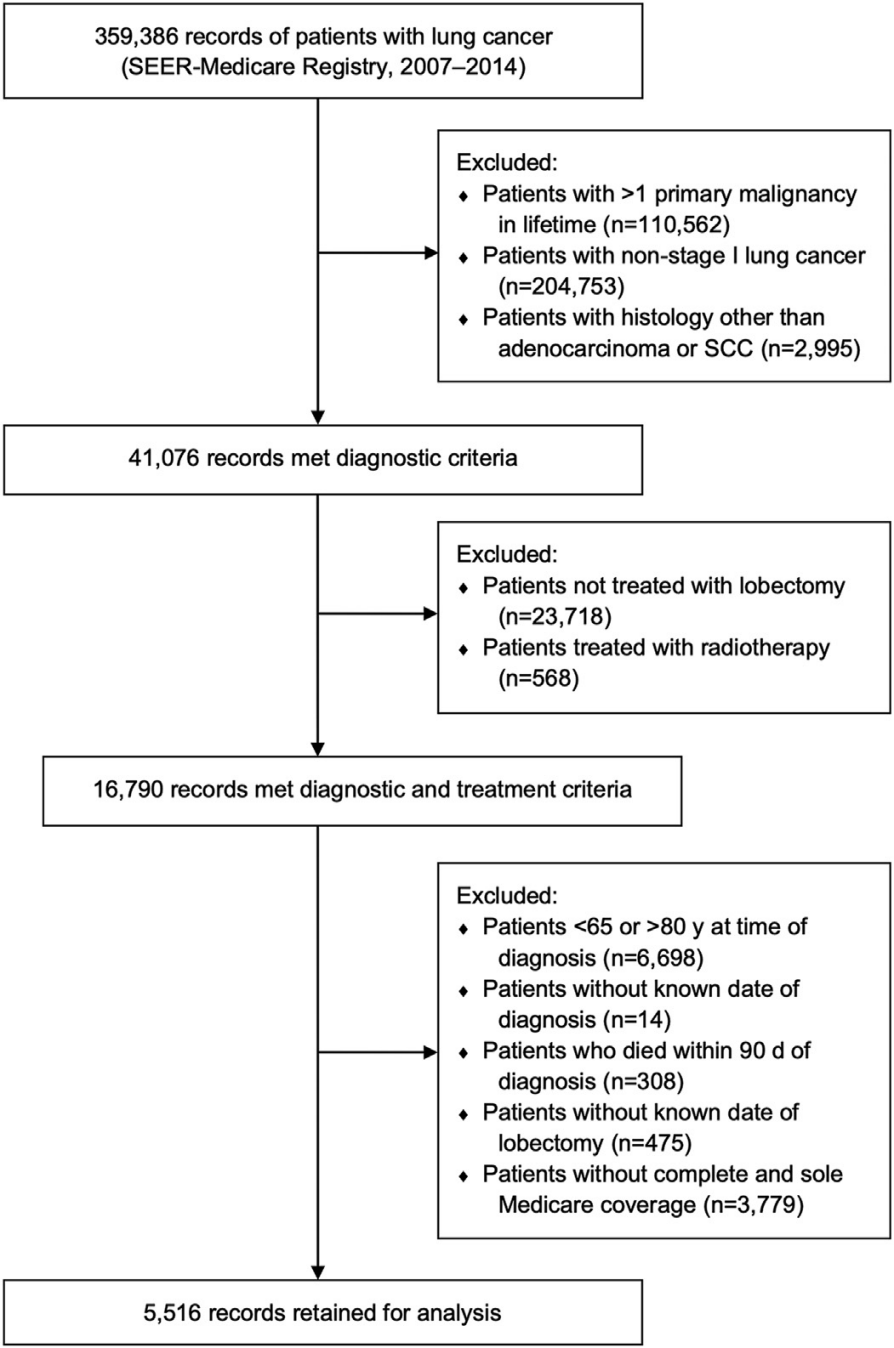
Consolidated Standards of Reporting Trials (CONSORT)-like flow diagram depicting inclusion and exclusion criteria. (SEER, Surveillance, Epidemiology, & End-Results; SCC, squamous cell carcinoma.)

A total of 5516 patients fulfilled inclusion criteria for this study (Figure 1). Of these, 1369 (24.8%) patients had 1 or more psychiatric comorbidities. Demographic and clinical characteristics of the study population are presented in Table 1.

**Table 1:** Demographic Characteristics

Characteristic	Patients With PC (n = 1369)	Patients Without PC (n = 4147)	Entire Study Population (N = 5516)	*P* Value^d^
Age, y				**<.001**
Mean	71.8	72.6	72.4	
Median	71	72	72	
Range	66–80	66–80	66–80	
Sex				**<.001**
Female	915 (66.8)	2196 (53.0)	3111 (56.4)	
Male	454 (33.2)	1951 (47.0)	2405 (43.6)	
Race				**.005**
White	1244 (90.9)	3645 (87.9)	4889 (88.6)	
Black	66 (4.8)	230 (5.5)	296 (5.4)	
Other	59 (4.3)	272 (6.6)	331 (6.0)	
Stage				**<.001**
IA	813 (59.4)	2244 (54.1)	3057 (55.4)	
IB	556 (40.6)	1903 (45.9)	2459 (44.6)	
Histology				.087
Adenocarcinoma	892 (65.2)	2806 (67.7)	3698 (67.0)	
SCC	477 (34.8)	1341 (32.3)	1818 (33.0)	
Surgical approach^a^				.551
Open	841 (64.0)	2531 (64.1)	3372 (64.0)	
Nonopen	473 (36.0)	1420 (35.9)	1893 (36.0)	
Comorbidity score^b^				**<.001**
Mean	4.3	4.7	4.5	
Median	4	4	4	
Range	0–16	1–17	0–18	
PC diagnosis class^c^				
Any	1369	…	1369	
Depression	779 (35.9)	…	779 (35.9)	
Anxiety	705 (32.5)	…	705 (32.5)	
ADHD	6 (0.3)	…	6 (0.3)	
Bipolar disorder	50 (2.3)	…	50 (2.3)	
Schizophrenia	22 (1.0)	…	22 (1.0)	
OPD	86 (4.0)	…	86 (4.0)	
Affective disorder	250 (11.5)	…	250 (11.5)	
Alcohol use disorder	116 (5.3)	…	116 (5.3)	
Substance use disorder	92 (4.2)	…	92 (4.2)	

Values are presented as n (%) unless otherwise noted. Statistically significant variables are highlighted in bold.

ADHD, attention-deficit/hyperactivity disorder; OPD, other (non-schizophrenic) psychotic disorders; PC, psychiatric comorbidity; SCC, squamous cell carcinoma.

a Surgical approach data were unavailable for 251 patients (55 with PC, 196 without PC).

b Charlson Comorbidity Index, ranging from 0 to 36, calculated 1 year prior to date of lobectomy.

c Reflects percentage of total psychiatric diagnoses as some patients have multiple comorbidities.

d Pearson’s chi-square test; independent samples *t*-test.

**Figure 2:**
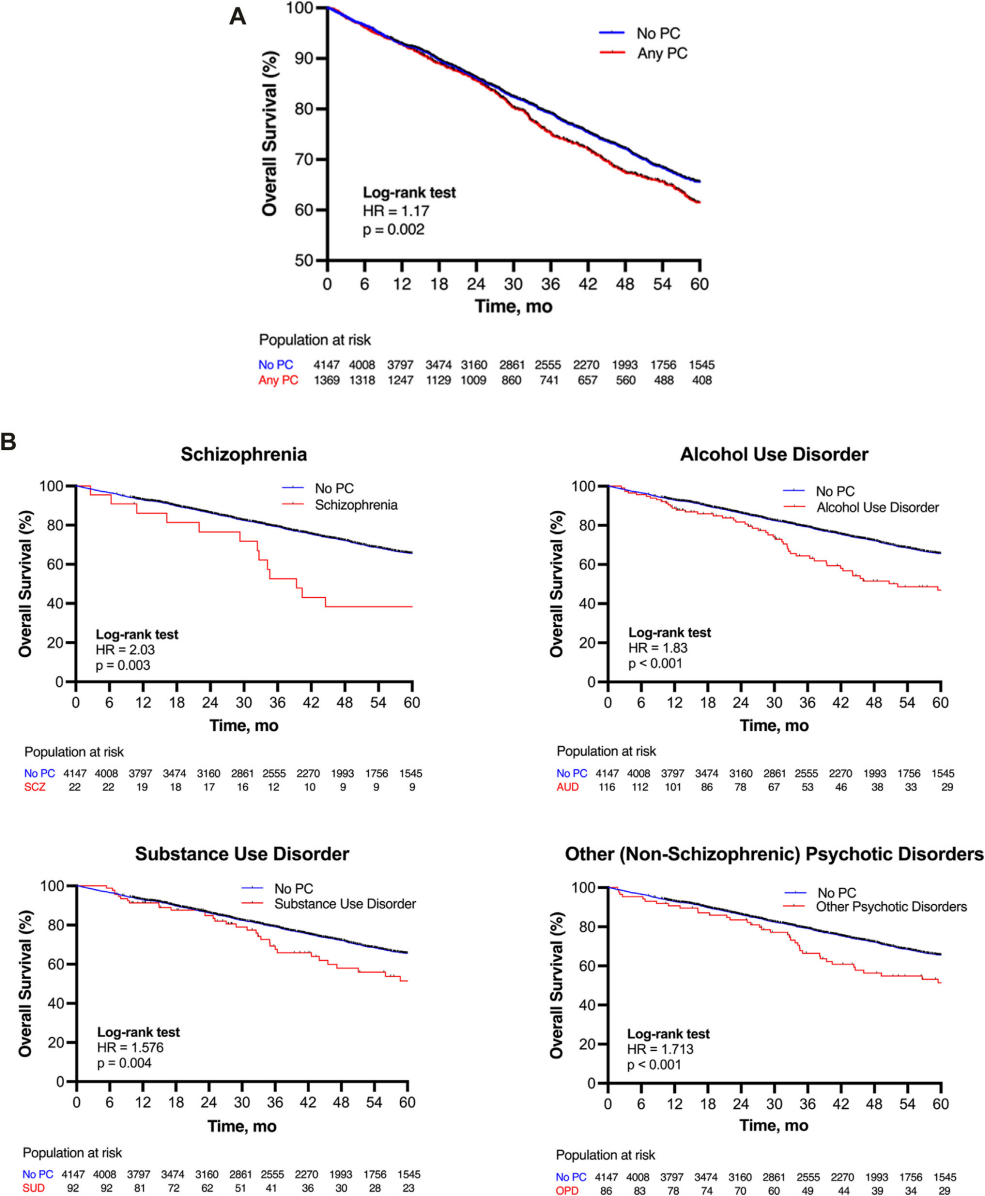
(A) Overall survival after lobectomy of patients with and without psychiatric comorbidities (PCs). (B) Psychiatric comorbidities significantly associated with overall survival in univariable analyses (AUD, Alcohol Use Disorder; OPD, Other Psychotic Disorders; SCZ, Schizophrenia; SUD, Substance Use Disorder).

On univariable analysis, having a preoperative diagnosis of any PC was associated with reduced overall survival at 5-year follow-up (hazard ratio, 1.17; 95% CI, 1.06-1.29) (Figure 2A). Age, male sex, white and black race, open lobectomy, Charlson Comorbidity Index, stage IB cancers, and squamous cell carcinoma were also associated with shorter survival (Table 2). When analyzing each PC independently, schizophrenia, nonschizophrenic psychotic disorders, alcohol use disorder, and substance use disorder were associated with shorter overall survival (Figure 2B). Anxiety, depression, attention-deficit/hyperactivity disorder, bipolar disorder, and nonspecified affective disorders were not significantly associated with overall survival.

**Table 2:** Results of univariable and multivariable analyses for overall survival

Characteristic	Overall Survival	
	Univariable Hazard Ratio (95% CI)	Multivariable Hazard Ratio (95% CI)
Age	**1.05 (1.04–1.06)**	**1.04 (1.03–1.05)**
Sex		
Female	**0.63 (0.58–0.68)**	**0.72 (0.66–0.78)**
Male	Ref	Ref
Race		
Caucasian	**1.59 (1.21–2.09)**	**1.49 (1.13–1.96)**
African American	**1.45 (1.18–1.80)**	**1.49 (1.21–1.85)**
Other	Ref	Ref
Stage		
IA	**0.65 (0.60–0.71)**	**0.74 (0.66–0.80)**
IB	Ref	Ref
Histology		
Adenocarcinoma	**0.63 (0.57–0.68)**	**0.81 (0.74–0.88)**
SCC	Ref	Ref
Surgical approach		
Open	Ref	Ref
Non-open	**0.75 (0.61–0.93)**	**0.78 (0.63–0.97)**
Comorbidity score^a^	**1.21 (1.19–1.23)**	**1.19 (1.17–1.20)**
PC diagnosis class^b^		
Any	**1.17 (1.06–1.29)**	**1.10 (1.05–1.15)**
Depression	1.14 (1.00–1.29)	Not included
Anxiety	1.09 (0.96–1.24)	Not included
ADHD	1.15 (0.29–4.58)	Not included
Bipolar disorder	1.38 (0.90–2.13)	Not included
Schizophrenia	**2.03 (1.26–3.27)**	**2.48 (1.53–4.00)**
OPD	**1.71 (1.30–2.27)**	Not included
Affective disorder	1.22 (0.99–1.51)	Not included
Alcohol use disorder	**1.83 (1.41–2.38)**	**1.45 (1.11–1.88)**
Substance use disorder	**1.58 (1.15–2.16)**	Not included

Statistically significant variables are highlighted in bold. ADHD, attention-deficit/hyperactivity disorder; OPD, other (non-schizophrenic) psychotic disorders; PC, psychiatric comorbidity; SCC, squamous cell carcinoma.

a Charlson Comorbidity Index, ranging from 0 to 36, calculated 1 year prior to date of lobectomy.

b Reflects percentage of total psychiatric diagnoses as some patients have more than 1 comorbidity.

Multivariable regression modeling adjusted for age, sex, race, cancer stage, cancer histology, surgical approach, Charlson Comorbidity Index, and presence of any psychiatric comorbidity retained PC in the final model (adjusted hazard ratio, 1.10; *P* < .001). Subgroup analyses demonstrated that this difference in mortality was driven primarily by the presence of schizophrenia (adjusted hazard ratio, 2.48; *P* < .001) and alcohol use disorder (adjusted hazard ratio, 1.45; *P* = .006). Substance use disorder (*P* = .266) and other nonschizophrenic psychotic disorders (*P* = .077), which were associated with overall survival in univariable analysis, were not significantly associated with survival in the final multivariable model.

## Comment

Previous studies have reported that patients with PCs experience more complications, longer hospital stays, and higher hospital readmission rates in the 30-day period after pulmonary lobectomy for NSCLC.^1^, ^2^, ^3^ This analysis demonstrates that patients with PCs experience decreased survival in the 5-year period after lobectomy for stage I NSCLC. The survival difference between patients with and without PC appears first at 30 months after lobectomy.

Potential explanations for the survival difference among patients with PC include lifestyle factors, chronic stress–related changes in immune response, and patient-provider communication challenges.^5^, ^6^, ^7^ In this study, schizophrenia and alcohol use disorder were significantly associated with reduced survival on multivariable analysis. The life expectancy of people diagnosed with alcohol use disorder is 24 to 28 years shorter than those without the diagnosis, which may contribute to the decreased survival observed in this cohort.^8^ Patients with schizophrenia often have additional comorbidities, altered symptom perception, and communication challenges that may impact their surgical outcomes.^9^

The findings from this retrospective observational study rely on the accuracy and availability of information in the SEER-Medicare database. While the SEER-Medicare database is reliable and highly curated, previous authors have noted its limitations, including that not all health data are captured in Medicare claims files and some diagnosis data may be incomplete.^10^ Because PCs in this study were identified by ICD coding, it is possible that noncoded PC diagnoses may have contributed to underreporting of patients with PC. It is also possible that the ICD codes in a patient’s claim data were historical and not representative of the patient’s mental state at the time of treatment.

This study only included patients aged 65 to 80 years with stage I adenocarcinoma or squamous cell carcinoma, limiting generalizability to other stages, histologies, and age groups. Strengths of this study include the large sample size, comprehensive multiinstitutional data, and its design that limits the effects of clinical stage, cancer histology, presence of other cancers, and response to nonsurgical treatments on overall survival. Further investigation is warranted to understand how the severity of a PC may impact mortality following lung cancer resection and if there are significant differences in causes of death between patients with and without PCs.

In summary, this large, population-based database analysis demonstrated that patients with PCs have reduced survival after lobectomy for early-stage NSCLC. Subgroup analyses revealed that this was driven by differences in survival of patients with schizophrenia and alcohol use disorder. Although the results are not designed to show the mechanistic basis of this observation, they highlight the need for targeted interventions and support for this vulnerable patient subgroup. Cancer care teams should consider preoperative counseling, psychiatric consultation, and ensuring adherence to medication regimens for patients with psychiatric comorbidities to optimize postoperative outcomes.
